# Targeting ferroptosis opens new avenues in gliomas

**DOI:** 10.7150/ijbs.96476

**Published:** 2024-09-03

**Authors:** Yuxin Wei, Yang Xu, Qian Sun, Yu Hong, Shanwen Liang, Hongxiang Jiang, Xinyi Zhang, Shenqi Zhang, Qianxue Chen

**Affiliations:** Department of Neurosurgery, Renmin Hospital of Wuhan University, Wuhan, 430060, Hubei, China.

**Keywords:** Gliomas, Ferroptosis, Molecular mechanism, Tumor immune microenvironment, TMZ resistance

## Abstract

Gliomas are one of the most challenging tumors to treat due to their malignant phenotype, brain parenchymal infiltration, intratumoral heterogeneity, and immunosuppressive microenvironment, resulting in a high recurrence rate and dismal five-year survival rate. The current standard therapies, including maximum tumor resection, chemotherapy with temozolomide, and radiotherapy, have exhibited limited efficacy, which is caused partially by the resistance of tumor cell death. Recent studies have revealed that ferroptosis, a newly defined programmed cell death (PCD), plays a crucial role in the occurrence and progression of gliomas and significantly affects the efficacy of various treatments, representing a promising therapeutic strategy. In this review, we provide a comprehensive overview of the latest progress in ferroptosis, its involvement and regulation in the pathophysiological process of gliomas, various treatment hotspots, the existing obstacles, and future directions worth investigating. Our review sheds light on providing novel insights into manipulating ferroptosis to provide potential targets and strategies of glioma treatment.

## 1. Introduction

Glioma is the most common primary malignant tumor in the central nervous system, with a poor prognosis and disappointing cure rates. Despite its low incidence, the short median overall survival (OS) time and high recurrence rate still make it one of the most challenging intracranial malignancies^1^, ^2^. Among all gliomas, glioblastoma multiforme (GBM) exhibits the most malignant phenotypes, including infiltrative growth, therapeutic resistance and high recurrence rate, and the worst prognosis^2^. Although the conventional standard for treatment involves maximum tumor resection, concurrent chemotherapy, and radiotherapy, the prognosis of glioma patients still remains dissatisfactory^3^, ^4^. Even though new therapeutic approaches, such as molecular targeted therapy, immunotherapy and tumor-treating fields have emerged, none of these treatments have provided reliable results for patients^5^, ^6^. The field of gliomas treatment still confronts with many obstacles, including chemotherapy resistance, tumor stem cells, and the tumor microenvironment^7^, ^8^. Triggering tumor cell death remains an indispensable strategy for treating glioma^9^, ^10^. Unfortunately, mounting evidence has unveiled that gliomas exhibit varying extent of tolerance to them, giving rise to frustration of many administrations^11^, ^12^.

Ferroptosis is a complex pathophysiological process that has recently been identified as a reactive iron-induced excess lipid peroxidation-dependent PCD^13^. The restraining of ferroptosis relies on iron/lipid metabolism homeostasis and functional ferroptosis defending systems, which provides valuable targets worth manipulating^14^. Genetically or pharmacologically inducing ferroptosis to trigger cell death has achieved excellent breakthrough in suppressing tumor progression, shedding light on the tricky field of gliomas^15^, ^16^. Unfortunately, the strong adaptability and flexibility of tumor cells may enable them to escape from ferroptotic death through intricate modulation mechanisms. Under certain circumstances, ferroptosis within tumors may even promote long-term tumor development^17^. Herein, we provide a review of the latest advancement in ferroptosis, summarize the mechanisms through which ferroptosis participates in glioma-related malignant phenotypes and more meaningfully, elaborate the limitations of previous studies and propose potential areas for future research, in an attempt to put forward new strategies for more accurate modulation of ferroptosis in gliomas, thus hindering its progression and even achieving cure, and improving the quality of patients' life.

## 2. The molecular mechanism of ferroptosis

**Figure 1:**
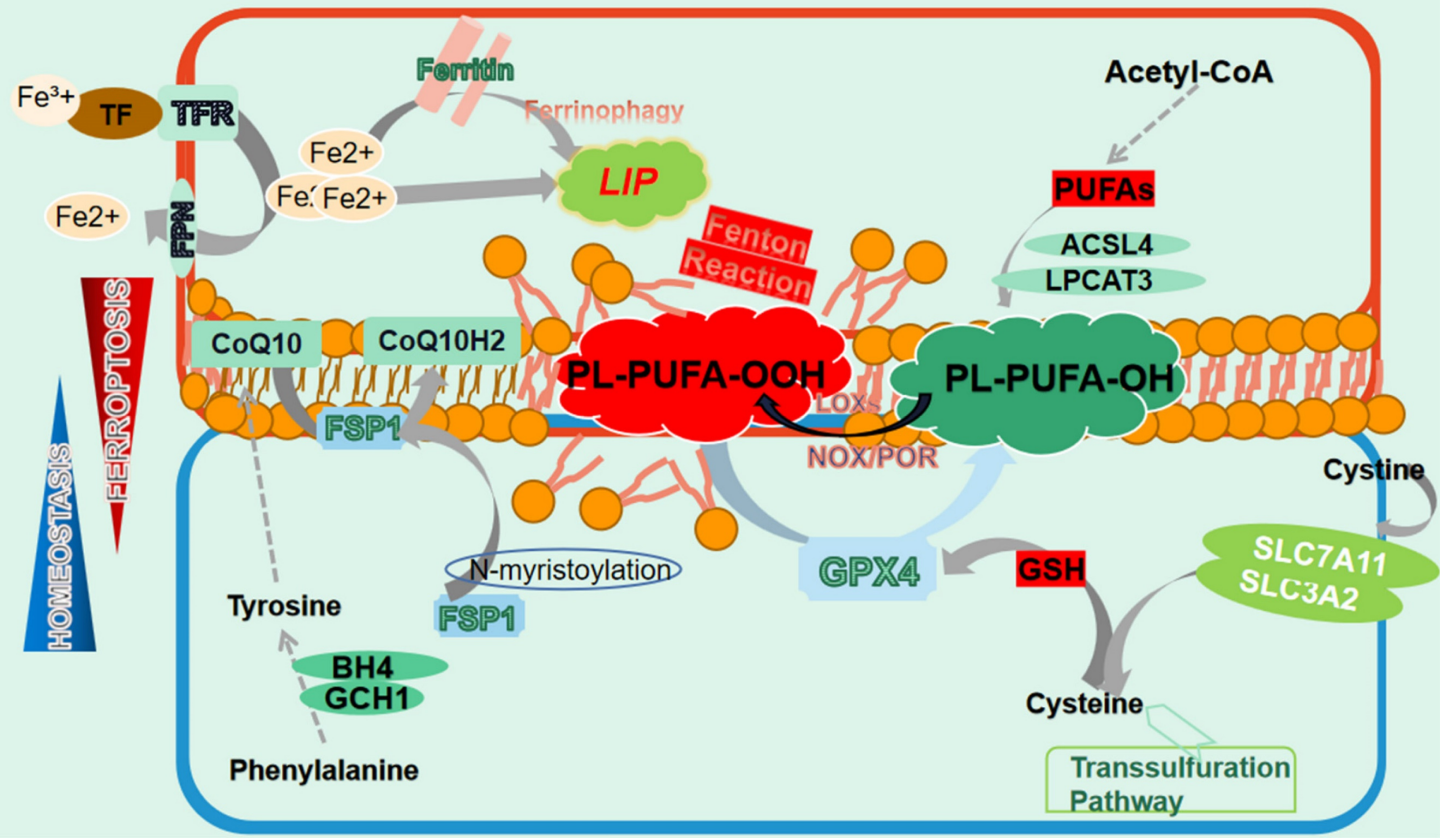
** The manifestation of ferroptosis.** Ferroptosis is precisely manipulated within cells whose occurrence relies on iron redundance, PUFAs peroxidation and the disturbance of antioxidant systems. Free iron from import and ferritin degeneration forms a LIP that initiates the Fenton reaction with PUFAs-containing PLs, which constitutes the ultimate ferroptosis executor and leads to cell membrane destruction. Nonetheless, several ferroptosis defending axis have been well-characterized, including the xCT-GSH-GPX4 axis, the FSP1-CoQ10-HADPH axis, the GCH1/BH4 axis as well as the DHODH-CoQ10 axis. Any perturbation in these pathways contributes to the development of ferroptosis. Additionally, the transsulfuration pathway is also involved in ferroptosis modulation.

Ferroptosis was firstly defined by Stockwell as a novel cell death modality distinct from apoptosis. It is manifested as iron redundancy, polyunsaturated fatty acid (PUFA) peroxidation, and abundant generation of lipid reactive oxygen species (ROS)^13^. Subsequent studies have provided more comprehensive understanding of this unique PCD^14^. Morphologically, ferroptotic cells display a contracted mitochondrial outer membrane, denser membrane structure, and a lack of mitochondrial cristae, without apoptotic vesicles or nuclear fragmentation^18^ (Fig. 1).

### 2.1 Lipid metabolism

Ferroptosis necessitates the presence of PUFAs, which contain easily extractable diallyl hydrogen atoms that are prone to lipid peroxidation^14^. This process is mediated by active iron, which converts lipid peroxides into alkyl free radicals that interact with adjacent PUFAs, triggering a chain reaction that eventually culminates in ferroptosis^19^. Unexpectedly, it is PUFAs-membrane phospholipids rather than free PUFAs that induce ferroptosis, highlighting the significance of PUFAs membrane integration. In fact, PUFAs synthesized under the action of acyl-CoA synthetase long-chain family member 4 (ACSL4) are transesterifified into PL-PUFAs-CoA via lysophosphatidylcholine acyltransferase 3 (LPCAT3), which are then oxidized to PL-PUFAs-POOL and ultimately cause cell membrane destruction and cell death^20^, ^21^. Intriguingly, although various membrane phospholipids have been reported to undergo ferroptosis, it is the PUFAs conjugated with them that determine the occurrence of ferroptosis^22^. Lipid transportation is also important for ferroptosis, with fatty acids transported into cells by fatty acid translocase potentially contributing to ferroptosis, while the release of oxidized PUFA tails from PLs mediated by phospholipase A2 group VI (iPLA2β) prevented this process^23^, ^24^. Peroxidation of PL-PUFAs-CoA relies on the involvement of a series of enzymes, represented by lipoxygenase (LOXs), which have the capacity of adding oxygen atoms to carbon atoms at specific positions of PUFAs^25^. Notably, LOXs' catalysis of membrane PL-PUFAs may require the assistance of other molecules. For instance, phosphatidylethanolamine binding protein 1 (PEBP1) can form a complex with 15-LOX to ensure that it acts on PE-PUFAs to ignite ferroptosis^26^. Additionally, both NADPH oxidase (NOXs) and P450 (cytochrome) oxidoreductase (POR) have been documented to facilitate lipid peroxidation in an NADPH-dependent manner^27^, ^28^. Moreover, hydroxyl radicals generated from the reaction between free ferrous iron and hydrogen peroxide can oxidize PUFAs by capturing hydrogen atoms at the diallyl site of PUFAs, representing an enzyme-independent autoxidation^29^.

### 2.2 Iron metabolism

Iron involves in many physiological processes and plays an indispensable role in cellular homeostasis^30^. However, excess intracellular iron can cause cell destruction by catalyzing ferroptosis. Iron absorption, transport and storage are precisely regulated to maintain a balance, where any disruption is likely to exacerbate the process of ferroptosis^31^. Iron from dietary and hemoglobin degradation binds to transferrin (TF) to form a TF-Fe3+ complex, which enter cells by interacting with transferrin receptor (TFR) on the membrana^32^, ^33^. Inside the cell, Fe^3+^ is reduced to Fe^2+^ via six-transmembrane epithelial antigen of prostate 3 (STEAP3) and released into the cytoplasm^34^. These Fe^2+^ is either stored as ferritin or transported out of the cell via ferroportin (FPN), while excess Fe^2+^ assembles as an unstable labile iron pool (LIP) in the cytoplasm^35^, ^36^. Due to its capability of transferring electrons to oxygen, iron bivalent ions trigger the Fenton reaction in the presence of hydrogen peroxides and produces substantial amount of ROS to trigger lipid peroxidation, eventually leading to ferroptosis and indicating that to some extent LIP determines cells' sensitivity to ferroptosis^37^, ^38^.

Iron chelators or ferric ammonium citrate affect ferroptosis by directly altering intracellular iron content by chelating or supplementing iron^39^, ^40^. Overexpression of TFR enhances the entrance of TF-Fe^3+^ complex into cells, thus facilitating ferroptosis, which is greatly mitigated by TFR silence^41^, ^42^. Iron responsive element binding protein 2 (IREB2), a crucial transcriptional regulator of iron metabolism, gives rise to accumulation of the LIP and lipid peroxidation, which boosts ferroptosis^43^. Conversely, nuclear receptor coactivator 4 (NCOA4)-orchestrated autophagic degradation of ferritin, known as ferritinophagy, exacerbates ferroptosis by liberating iron^44^. Hepcidin, a major regulator of iron homeostasis, contributes to ferroptosis by mediating the internalization and breakdown of FPN, leading to a reduction in iron export^45^. Nonetheless, controversies persist as hepcidin administration has been observed to unconventionally alleviate iron accumulation^46^. Recent studies have also demonstrated that some microvesicles directly expelled ferritin as exocytosis^47^, ^48^.

### 2.3 Anti-ferroptosis mechanisms

#### 2.3.1 xCT-GPX4 axis

Cumulative studies have highlighted the significant role of xCT- glutathione peroxidase 4 (GPX4) axis in resisting ferroptotic stress^49^. System Xc-, also known as xCT, is a widely distributed transmembrane protein composed of functional subunit solute carrier family 7 member 11 (SLC7A11) and regulatory subunit solute carrier family 3 member 2 (SLC3A2) and is responsible for reverse transport of intracellular glutamate and extracellular cystine^50^, ^51^. Cystine is subsequently converted to cysteine and synthesized into glutathione (GSH) by glutamate cysteine ligase (GCL) and glutamylcysteine synthetase (GCS), which defines GPX4 activity^52^, ^53^. As the superior ferroptosis regulator, GPX4 predominantly converts PUFA-PL-OOH to corresponding alcohol derivatives by utilizing GSH, which restrains ferroptosis and maintains intracellular homeostasis^54^, ^55^.

The modulation of xCT significantly affects cell ferroptosis. p53 ignites ferroptosis by mediating SLC7A11 prohibition, which is one of the signals of its tumor suppressor effect^56^. In the tumor microenvironment, TNFγ downregulates SLC7A11 to activate CD^8+^ T cell ferroptosis, which facilitates T cell exhaustion and thus contributes to tumor cell survival^57^. On the contrary, OTU domain-containing ubiquitin aldehyde-binding proteins otubain 1 (OTUB1) suppresses xCT ubiquitination, enabling cells tolerant to ferroptosis^58^. Furthermore, various regulators have also been elucidated involved in ubiquitination-mediated ferroptotic modulation, providing potential pharmacological therapeutic targets^59^, ^60^. GPX4 is also subject to multiple modulation to interfere with the process of ferroptosis^54^. The first discovered GPX4 inhibitor, RSL3, covalently binds to GPX4, bringing about its inactivation and the accumulation of intracellular peroxidized PUFAs^61^. Numerous compounds, including FIN56 and FINO2, induce lipid peroxidation and cell death by manipulating GPX4^62^, ^63^. Moreover, transcription factor 4 (TCF4) binds to the GPX4 promoter region and translationally upregulates GPX4, thereby leading to impeded ferroptosis^64^, ^65^.

Since GPX4 is a selenium-containing protein, a selenium-rich diet also appears to alleviate the damage caused by ferroptosis in a mouse intracranial hemorrhage model by upregulating GPX4^66^, ^67^. Furthermore, 2-Cyano-3,12-dioxooleana-1,9 (11)-dien-28-oic acid prevents GPX4 degradation by intervening with chaperone-mediated autophagy, thereby halting cell ferroptosis^68^.

#### 2.3.2 FSP1-CoQ10-NADPH axis

Several tumor cells insensitive to GPX4 inhibitors led to the identification of the non-classical ferroptosis defensive FSP1-CoQ10-NADPH pathway^69^, ^70^. The ferroptosis suppressor protein 1 (FSP1), previously known as apoptosis-inducing factor 2, presents as a novel ferroptosis regulator. Indeed, studies have indicated that FSP1 is an NADH-dependent oxidoreductase of CoQ, which reduces ubiquinone to panthenol, a lipophilic radical-trapping antioxidant (RTA) to halt the membrane lipid peroxidation^71^, ^72^. Notably, an N-terminal consensus sequence on FSP1 is pivotal for N-myristoylation and recruits it to cell membrane, where FSP1 executes its capability of mitigating lipid peroxidation^69^, ^72^. In addition, ubiquitination- and phase separation-mediated FSP1 regulation opens novel avenues through which the manipulation of ferroptotic cell death becomes more achievable^73^, ^74^. Furthermore, recent research has demonstrated that by reducing vitamin K to VKH2, a widely accepted RTA, FSP1 powerfully mitigates ferroptotic stress through maintaining the vitamin K cycling, providing a new prospect for ferroptosis modulation^75^. Notably, complicated structural analysis of FSP1 offers the possibility of developing specific targeted medicine^76^.

#### 2.3.3 DHODH-CoQ axis

In analogy to FSP1, the capacity of dihydroorotate dehydrogenase (DHODH) to obliterate ferroptosis is reliant on ubiquinone. Since DHODH is exclusively located on the outer side of mitochondrial inter membrane, it only addresses lipid peroxidation within mitochondria^77^. Mitochondria-derived significant amount of ROS during oxidative phosphorylation results in vulnerability to lipid peroxidation in the presence of mitochondrial antioxidant system disturbance^78^. However, DHODH generates an ample quantity of CoQ10 while simultaneously oxidizing dihydroorotate, which effectively eliminates lipid peroxides within mitochondrial^79^. Hijacked by breast tumor cells, the DHODH redox signal provides a powerful shelter against lipid peroxidation while blocking it by nanomaterials drastically strengthens ferroptotic cell death and suppresses tumor advancement^80^. Moreover, DHODH also relieves lipid peroxide production via the p53/ALOX15 signaling^81^.

#### 2.3.4 GCH1-BH4 axis

GTP cyclohydrolase 1 (GCH1)-BH4 is demonstrated highly expressed in cancer cells tolerant to ferroptosis^82^. Previous studies have unveiled that tetrahydrobiopterin/dihydrobiopterin (BH4/BH2) rescue cell viability while silence of dihydrofolate reductase (DHFR), coupled with GPX4 inhibition, synergizes to facilitate ferroptosis^83^. Considering that on one hand, CoQ10 has been identified to elevate following GCH1 overexpression, on the other hand, BH4 catalyzes the synthesis of 4-OH-benzoate, a precursor to CoQ10, we draw the hypothesis that the GCH1-BH4 axis may act to hinder ferroptosis by boosting the generation of CoQ10^84^. Intriguingly, blockage of GCH1/BH4 axis unexpectedly triggers the activation of ferritinophagy, implying an interaction between GCH1/BH4 and iron metabolism^85^. Moreover, a lipid omics investigation demonstrates that the GCH1-BH4 pathway selectively influences phosphatidylcholines containing two PUFA chains, such as PC20:4_20:4, PC 20:4_20:5, and PC 20:4_22:6, although further exploration is warranted^84^.

Overall, it is plausible to propose that the manifestation of ferroptosis is contingent on the disruption of both iron and lipid metabolism, as well as the decline of the intracellular antioxidant systems. Furthermore, several ferroptosis defending axis have been identified and characterized, which function in diverse cell species, organelles, or cell subregions to maintain the intracellular REDOX balance^14^, ^86^. Nonetheless, a latest report may consider amino acid oxidase interleukin-4-induced-1 (IL4i1) and indoleamine2,3-dioxygenase1 (IDO1) as novel regulators suppressing ferroptosis by generating the metabolite indole-3-pyruvate^87^, ^88^.

## 3. Targeting ferroptosis in gliomas

Enormous attempts have emerged to restrain tumor advancement by inducing cell death, including apoptosis, pyroptosis, necroptosis and others. In terms of gliomas, however, the effectiveness of these approaches is frustrated^89^, ^90^. Given that ferroptosis has been implicated in various biological processes of gliomas, significant effort has been devoted to interfering with the crucial ferroptosis-related signals in order to affect malignant phenotypes in gliomas.

### 3.1 Modulation of metabolic pathways in gliomas

#### 3.1.1 Lipid metabolism

Lipid metabolism in glioma cells is distinct from that in normal cells. A high quantity of lipids that make up the phospholipid bilayer is in demand by gliomas to sustain their unlimited proliferation. Moreover, numerous intricate signals favoring tumor cells are often implemented by altered lipid metabolism^91^. There is no doubt that lipid metabolic reprogramming, especially that of PUFAs, plays a significant role in glioma ferroptosis.

As previously mentioned, ACSL4-mediated PUFAs synthesis and membrane integration is vital for the initiation of ferroptosis, for which it has garnered significant attention concerning glioma management. It has been demonstrated that gliomas notably reduce ACSL4 expression^92^. By elevating miR-670-3p that targets ACSL4, gliomas render resistance to ferroptotic stress^93^. A convey revealing the relationship between ferroptosis-related genes (FRGs) and gliomas indicated that interferon regulatory factor 2 (IRF2) was positively associated with glioma grade and contributed to gliomas, potentially by maintaining low ACSL4 level and high xCT/GPX4 level^94^. Nonetheless, activated HIF2a by Roxadustat improves several lipid-related genes, including ACSL4, and unexpectedly evokes ferroptosis in gliomas, which calls for further exploration^95^. Indeed, overexpression of ACSL4 in gliomas leads to ferroptosis and halted proliferation, as is observed when capsaicin is applied to augment ACSL4 level^92^, ^96^. Beyond that, heat-shock protein 90 (HSP90)-Drp1-mediated ACSL4 stabilization strengthens the anti-glioma activity of erastin^97^.

Notably, Murine Double Minute 2/X (MDM/MDMX) may reprogram the cellular lipid profile to favor ferroptosis via FSP1 manipulation^98^. And though ferroptosis in glioma cells is anticipated to impede its progression, a recent study has unveiled that recruited neutrophils during the early stages of GBM amplified GBM necrosis by transferring myeloperoxidase-containing granules into tumor cells and triggering lipid peroxides-dependent ferroptosis. Since tumor necrosis is typically associated with a poor prognosis, the study in fact unconventionally unveils a pro-tumorigenic role of ferroptosis in gliomas^17^.

Nonetheless, glioma represents a malignancy with high heterogeneity which the glioma lipidome also possesses, with specific fatty acids and saturation states in distinct subclasses. A recent report has documented that cyclin dependent kinase inhibitor 2A (CDKN2A) in GBM governs the sequestration of oxidizable PUFAs into triacylglycerides (TAGs) within lipid droplets and alters the acyl tail composition in fatty acids, enabling tumor cells more tolerant to lipid peroxidation^99^. CDKN2A depletion, however, significantly reduces PUFAs isolation into TAG pool, priming for enhanced lipid peroxidation and ferroptosis^99^. Therefore, identifying the heterogeneity of specific lipid regulatory molecules in different glioma subgroups may be a promising avenue for future research. It is worth noting that the distribution of lipids, especially PUFAs, may also be a potential target for modulating ferroptosis in gliomas.

#### 3.1.2 Iron metabolism

The malignant phenotype of glioma cells, including infinite proliferation, poses a higher demand for iron than normal cells, which also indicates higher susceptibility to ferroptosis^100^. Moreover, glioma cells have been observed to possess more iron content than other brain tumors, which may be attributed to abnormal expression of TFR^101^. Pseudolaric acid B has the ability to upregulate TF and its receptor to enhance iron import, thus activating NOX4 and arousing ferroptosis in glioma cells^102^. In contrary, glioblastoma-mediated iron export via miR-147a-SLC40A1 (encoding FPN) axis is conducive to the elimination of ferroptosis^103^. And canonical temozolomide may also drive gliomas growth inhibition partially by facilitating divalent metal transporter 1 (DMT1)-mediated iron transport^104^. Notably, taking advantage of TF, the compositely-designed TF-modified nanoparticles loaded with asiatic acid has exerted improved targeting and excellent anti-glioma efficiency^105^. Moreover, the abnormal expression of STEAP3 in gliomas may also serve as a valuable prognostic marker, which not only has an impact on TFR, but also tightly correlates with patients' prognostic with gliomas^106^, ^107^.

Within the cell, iron beyond requirement is either stored as ferritin or utilized to form an LIP. Ferritin autophagic degeneration, named ferritinophagy, inversely releases iron into cytoplasm to enhance the ferroptosis vulnerability. Dihydroartemisinin (DHA) effectively suppresses gliomas by promoting ferroptosis. However, activated lncRNA TUG1-Myc-associated zinc finger protein (MAZ)-mediated heavy ferritin chain (FTH) upregulation attenuates its efficiency^108^. In contrast, Amentoflavone can facilitate ferroptotic death in gliomas by enhancing FTH degeneration^109^. Additionally, NCOA4 serves as a pivotal cargo mediating ferritinophagy. When prohibited by Tripartitemotif (TRIM), as well as matrix remodeling-associated protein 8 (MXRA8), through ubiquitination or other uncoined mechanisms, GBM gets potentiated due to alleviated ferritin degeneration and ferroptosis^110^, ^111^. In contrast, the ALDH1a3 (Aldehyde dehydrogenase 1 family member A3)-LC3B complex markedly sensitizes GBM to RSL3^112^.

All the aforementioned studies have sufficiently illustrated the significance of ferritin, particularly ferritinophagy, in the ferroptosis of glioma cells. As a matter of fact, quite a few nanomaterials have successfully suppressed glioma by modifying ferritin or supplying an overwhelming amount of iron intracellularly, providing new approaches for glioma treatment^113^.

### 3.2 Modulation of anti-ferroptosis axis in gliomas

#### 3.2.1 xCT-GSH-GPX4 axis

The canonical anti-ferroptosis pathway eliminates ferroptotic stress dependent on xCT stability, GSH content and GPX4 activity^53^, whose alteration occurs prevalently in gliomas^54^.

xCT locates upstream of the moderation of ferroptosis in gliomas by managing cysteine import and subsequent GSH synthesis. Transcriptionally, the m6A reader NF-κB activating protein (NκAP) promotes SLC7A11 mRNA splicing and maturation by recruiting splicing factor proline and glutamine-rich (SFPQ)^114^. Post-transcriptionally, OTUB1 stabilizes xCT protein via direct binding^115^. Moreover, a proteomic analysis implies that Bach1 (BTB and CNC homology 1), highly expressed in gliomas, accelerates tumor invasion while conversely confers enhanced vulnerability to ferroptosis, possibly due to altered SLC7A11 expression^116^. Oppositely, enhanced IRF2 in gliomas may interfere with SLC7A11 and GPX4 to extinguish ferroptosis^94^. In addition, through modifying platelet derived growth factor receptor alpha (PDGFRA)-xCT, elevated circCDK14 notably reduces gliomas' sensitivity to ferroptosis^117^. Furthermore, thioredoxin domain protein 12 (TXNDC12) protects gliomas from ferroptotic stress by modulating xCT^118^.

By reducing the reduced GSH to its oxidized compartment, GPX4 converts cytotoxic PUFA-POOLs to derivative PUFA-OHs, thus impeding ferroptosis and maintaining cellular homeostasis. The first GPX4 inhibitor RSL3 has been proven effective in suppressing gliomas by stimulating GPX4 inactivity in a dose-dependent manner^119^. Interestingly, the combination of RSL3 and TMZ exerts even more pronounced effects on restraining gliomas, regardless of IDH WT/mutant, hinting at a potential clinical strategy^120^. FIN56 is an another classic ferroptosis inducer, where the graphdiyne nanoplatforms GDY-FIN56-RAP effectively promotes FIN56-mediated selective lysosomal degradation of GPX4, exhibiting potential anti-glioma efficacy^121^. Some natural extracts drastically trigger glioma ferroptosis and progression arrest by impacting on GPX4. Plumbagin and capsaicin present remarkable limitation on gliomas through either promoting lysosome-mediated GPX4 degeneration or decreasing its level^96^, ^122^. In addition, DHA also initiates lipid peroxidation and ferroptotic cell death in gliomas by targeting GPX4^123^. However, researchers have revealed that activated ER stress by DHA might in turn enhance the expression and activity of GPX4, which neutralizes the glioma-killing effect of DHA^124^. In accordance with this, sevoflurane activates the ER stress executor ATF4 to interfere with GPX4^125^. Lastly, the anti-cancer drug apatinib restrains gliomas partially through decreasing GPX4 and amplifying ferroptosis^126^.

It is significant to note that glioma cells may orchestrate GPX4 to favor their malignancies. For instance, elevated fragile X mental retardation 1 (FXR1) confers gliomas resistance to TMZ. Mechanistically, FXR1 binds with GPX4 mRNA to boost its expression, subsequently halting ferroptosis^126^. Beyond that, SLC1A5, which participates in immune modulation, is positively correlated with GPX4, though the underlying mechanism is ill-defined, suggesting a potential interaction between glioma immune microenvironment and GPX4^127^.

Theoretically, any perturbation to xCT affects the GSH content, while GSH, to some extent, determines GPX4 activity^102^. Furthermore, other pathways impacting GSH content are commonly present in gliomas. Some neuroprotectors, such as curcumin and melatonin, protect glioma cells against oxidative damage by strengthening glutamate-cysteine cigase catalytic subunit (GCLC) generation, which serves as a subunit of GCL catalyzing GSH synthesis^128^, ^129^. Besides, a genome editing system has been established targeting glutathione synthetase-mediated GSH synthesis and dramatically improves the radiosensitivity of gliomas by igniting ferroptosis occurrence^130^. Apolipoprotein C1 (APOC1)-mediated cystathionine beta-synthase increase heightens the transsulfuration pathway and ultimately enhances GSH production and GPX4^131^. Conversely, capsaicin and auranofin administration generate a decrease in GSH level and thereby foster glioma progression^96^, ^132^. Intriguingly, gamma-glutamyltransferase 1, an enzyme that cleaves extracellular glutathione, halts cystine deprivation-induced GSH depletion and ferroptosis in glioblastoma with high cell density, indicating a novel pathway mediating GSH-GPX4 axis distinct from cystine deprivation or inhibition of cystine uptake^133^.

#### 3.2.2 GPX4-independent pathways

It has become a consensus that there exists GPX4-independent anti-ferroptosis axis in cells, which has garnered attention in terms of synergizing with classical ferroptosis inducers in treating gliomas. Overexpressing CircLRFN5 in glioblastoma has been demonstrated to mediate paired related homeobox 2 (PRRX2) protein degradation via the ubiquitin-proteasomal pathway, which transcriptionally downregulates GCH1-BH4 in GSCs and provokes ferroptosis^134^. Intriguingly, fear stress is demonstrated to stabilize FSP1 mRNA through methyltransferase like 3-related m6A modification and therefore contributes to glioma progression^135^. Considering the potential crosstalk between olfactory sense and glioblastoma, it is reasonable to assume that both the endocrine and nervous systems may be involved in glioma ferroptosis^136^.

### 3.3 Non-coding RNAs

Non-coding RNAs refer to a cluster of RNAs that do not undergo translation and execute various biological functions through interacting with a wide range of proteins and mRNAs^137^. These non-coding RNAs play a significant role in the progression of gliomas, and recently, many studies have indicated their involvement in the outcome of ferroptosis in gliomas as well^138^. A study has established a ferroptosis-based lncRNA signature to predict the immune landscape and radiotherapy response in glioma patients, where 15 lncRNAs have been identified by the transcriptomic analysis at the aim of providing precise individualized therapeutic option^139^. Generally, lncRNAs appear to modulate diverse branches of ferroptosis. For instance, Taurine upregulated 1 (TUG1) targets MAZ and negatively regulates FTH1 expression, thereby strengthening the anti-glioma effect of DHA^108^. By recruiting adenosine deaminase acting on RNA (ADAR) to stabilize GLS2 mRNA, ATXN8OS is capable of triggering ferroptosis and restraining TMZ resistance^140^. Apart from that, elevated transmembrane protein 161B-AS1 facilitates the malignancies of glioma cells and TMZ tolerance, where it sponges hsa-miR-27a-3p to enhance the expression of ferroptosis-related genes like fanconi anemia complementation group D2 (FANCD2) and CD44^141^. In addition, cancer-associated fibroblasts-derived heat shock factor 1 transcriptionally increases lncRNA DLEU1, which upregulates SLC7A11 through mediating ATF3 mRNA degradation by binding with zinc finger protein 36 (ZFP36)^142^. LincRNAs, a specific subset of lncRNAs, also attract interest. Linc02381 is able to target the glucose transporter SLC2A10 in GBM to regulate ferroptosis via encoded micropeptides^143^. While through interacting with serine/arginine splicing factor 1 (SRSF1)/MAPK8, Linc01564 positively associates with Nrf2 and blocks ferroptosis in gliomas^144^.

Circular RNAs also participate in ferroptosis manipulation in gliomas. CircCDK14 indicates a poor prognosis for glioma patients. By sponging miR-3938, it upregulates the oncogenic gene PDGFRA and thus reduces glioma cells' sensitivity to ferroptosis^117^. Moreover, the upregulation of circTTBK2-triggered activation of miR-761/ integrin subunit beta 8 (ITGB8) hinders ferroptosis in glioma and facilitates tumor proliferation^145^. In addition, the GCH1/BH4 axis may as well be a target of circLRFN5 in ferroptosis resistance^134^.

It should be noted that many miRNAs, such as miR-147a and miR-670-3p, also possess the capacity of adjusting glioma ferroptosis by modifying iron and lipid metabolism^93^, ^103^. And targeting ferroptosis-related molecular via non-coding RNAs has proven a success for glioma suppression in nanomaterial therapy-based fields^113^.

### 3.4 Nrf2 and p53

Nrf2 is a vital regulator of intracellular redox biology and is maintained at a low level through keap1-mediated degradation^146^. Since ferroptosis-related genes including xCT, GPX4 and FTH1 present as targets of Nrf2, many treatments seek to disturb ferroptosis by manipulating Nrf2 in gliomas. Fostered Nrf2 expression obliterates ferroptosis by upregulating xCT, subsequently accelerating proliferation and oncogenic transformation in GBM. And so does keap1 silence^147^. Reinforced apolipoprotein C1 as well as Linc01564 in gliomas also fortifies the expression of ferroptosis protective genes HO-1, NQO1 and FTH1 via the keap1-Nrf2 axis^131^, ^144^. Beyond that, GSK3β mediates Nrf2 degeneration, whereas AKT suppresses the activity of GSK3β, which stabilizes Nrf2 to defend ferroptosis in IDH-mutated glioma^148^. Conversely, a study has revealed that high Nrf2 enables TMZ-resistant cells susceptible to ferroptosis. Mechanistically, activated adenosine triphosphate binding cassette subfamily C member 1 (ABCC1)/MRP1 by Nrf2 gives rise to the phenomenon known as collateral sensitivity in glioblastoma, where MRP1 channels export GSH and GSH-bonded TMZ out of cells, bringing about higher sensitivity to ferroptosis^149^. Furthermore, GSH synthesis can also be amplified by Nrf2-mediated GCLC and glutathione synthetase upregulation^150^. All above indicates that targeting Nrf2 in ferroptosis could be a promising therapeutic option to improve the outcome for glioma patients, although further investigation is desirable.

Similar to Nrf2, the well-known tumor suppressor p53, frequently mutated in cancers, also disrupts glioma progression by moderating ferroptosis. Notably, by directly depleting SLC7A11, p53 decreases the activity of xCT to boost ferroptosis^151^. The ubiquitination of p53 affects its capacity of modulating ferroptosis. RND1, the Rho family GTPase 1, a novel regulator of p53, can bind to and deubiquitinate p53, thus expurgating SLC7A11 and highlighting ferroptosis^152^. Conversely, E3 ubiquitin ligase MDM2 likewise mitigates ferroptotic responses through ubiquitination degradation of p53^153^. What's interesting is that the mutation status of p53 serves as a meaningful regulatory factor in ferroptosis. A study demonstrates that in p53 wild-type GBM, classical p62-mediated Nrf2 activation prominently regulates SLC7A11 expression and plays an anti-ferroptosis role. However, in mutant p53 GBM, the stronger interaction of mutant-p53/Nrf2, together with increased p53's transcriptional suppression on SLC7A11 mediated by p62, reverses the abovementioned inhibitory effect^154^. Additionally, other post-translational modifications like acetylation, which is pivotal in impacting p53' capacity of regulating ferroptosis are worth attention^155^. Lastly, p53 may also take a part in impacting LOXs-mediated lipid peroxidation^156^.

### 3.5 ER stress and NF-κB pathway

Endoplasmic reticulum (ER) is a vital organelle involved in the synthesis and transportation of normal proteins and lipids within cells. However, excessive accumulation of unfolded proteins under internal/external stimuli causes activated endoplasmic reticulum stress (ER stress), where persistent ER stress gives rise to cell death^157^. Emerging studies have affirmed the participation of both ER and ER stress in ferroptosis^158^. Compounds that modulate ferroptosis by affecting lipid peroxidation primarily localize to the ER and it is sufficient to block ferroptosis by suppressing lipid peroxidation in the ER, indicating that the ER is the central hub of lipid peroxidation during ferroptosis^14^. Moreover, the volume of ER may also be a key determinant of susceptibility to ferroptosis^14^, ^159^.

ER stress is primarily implemented via three executors namely IRE1α (inositol-requiring enzyme 1α), PERK (PKR-like endoplasmic reticulum kinase) and ATF6 (activating transcription factor 6), which ignites downstream signaling cascades. How ER stress modulates ferroptosis remains a topic of debate. First of all, ferroptosis inducers (erastin) simultaneously triggers ferroptosis and ER stress, whereas the latter in turn heightens ferroptosis occurrence^160^. Activated ATF4 downstream PERK by Sevoflurane enhances ChaC glutathione-specific gamma-glutamylcyclotransferase 1 (CHAC1) and elevates transferrin, ferritin as well as ROS levels, thereby leading to the advancement of ultimate ferroptosis in GBM cell lines^125^. By triggering ER stress, brucine-mediated ATF3 upregulation and entrance into nuclei disturbs NOX4, superoxide dismutase 1 and xCT to contribute to ferroptosis^161^. On the other hand, though, ER stress, especially the downstream gene ATF4 restricts the extent of ferroptosis. As a transcriptional factor, ATF4 mediates the upregulation of its target genes like xCT and GPX4. Indeed, ATF4 activation elevates xCT to confer ferroptosis tolerance while strengthening chemo-resistance^162^. Studies have also indicated that under DHA administration, activated ER stress neutralizes lipid peroxidation via PERK-ATF4-HSPA5-mediated GPX4 upregulation^124^. Moreover, excessive autophagy triggered by ER stress is also involved in ferroptosis modulation^163^. These findings suggest that the interplay between ER stress and ferroptosis is complex and even contradictory.

NF-κB signaling is involved in both ER stress and ferroptosis. The activation of NF-κB signaling by RSL3 is observed to be negatively correlated with ATF4 and xCT, indicating its role in suppressing ferroptosis^164^. However, the NF-κB inhibitor Myrislignan induces ferroptosis through EMT-dependent signaling^165^.

### 3.6 Autophagy

Autophagy is an evolutionally conserved biological process that degrades specific intracellular molecules and organelles for maintaining homeostasis in a lysosome-dependent manner. Increasing studies have considered ferroptosis as an autophagic cell death process, while inappropriate autophagy might hasten ferroptotic cell death^166^. Amentoflavone initiates ferroptosis by promoting autophagy via AMPK/mTOR signaling, while autophagy inhibitor BafA1 or depletion of ATG7 compromises this effect^109^. By enhancing the expression of autophagy-related protein Beclin1 and LC3II and suppressing that of p62, synthesized IONP@PTX possesses the capacity of triggering ferroptosis to confine GBM growth, whereas inhibition of autophagy dramatically weakens its efficacy^167^.

Particularly, ferritinophagy refers to the process of autophagic degradation of ferritin, which liberates free iron into cytoplasm, bringing about oxidative damage and eventual ferroptosis via the Fenton reaction. This process requires the assistance of a selective cargo receptor NCOA4. TRIM7-mediated NCOA4 ubiquitination and degeneration has been elaborated to alleviate ferroptosis and GBM' sensitivity to TMZ^111^. Conversely, MXRA8 protects gliomas from ferritin degradation-dependent ferroptosis through negatively regulating NCOA4 and upregulating FTH1, although the explicit mechanism remains uncertain^110^. Unexpectedly, overwhelming cytoplasmic iron can expedite the degradation of NCOA4 in a ubiquitin-proteasome dependent manner, supplying a negative feedback loop^168^.

**Figure 2:**
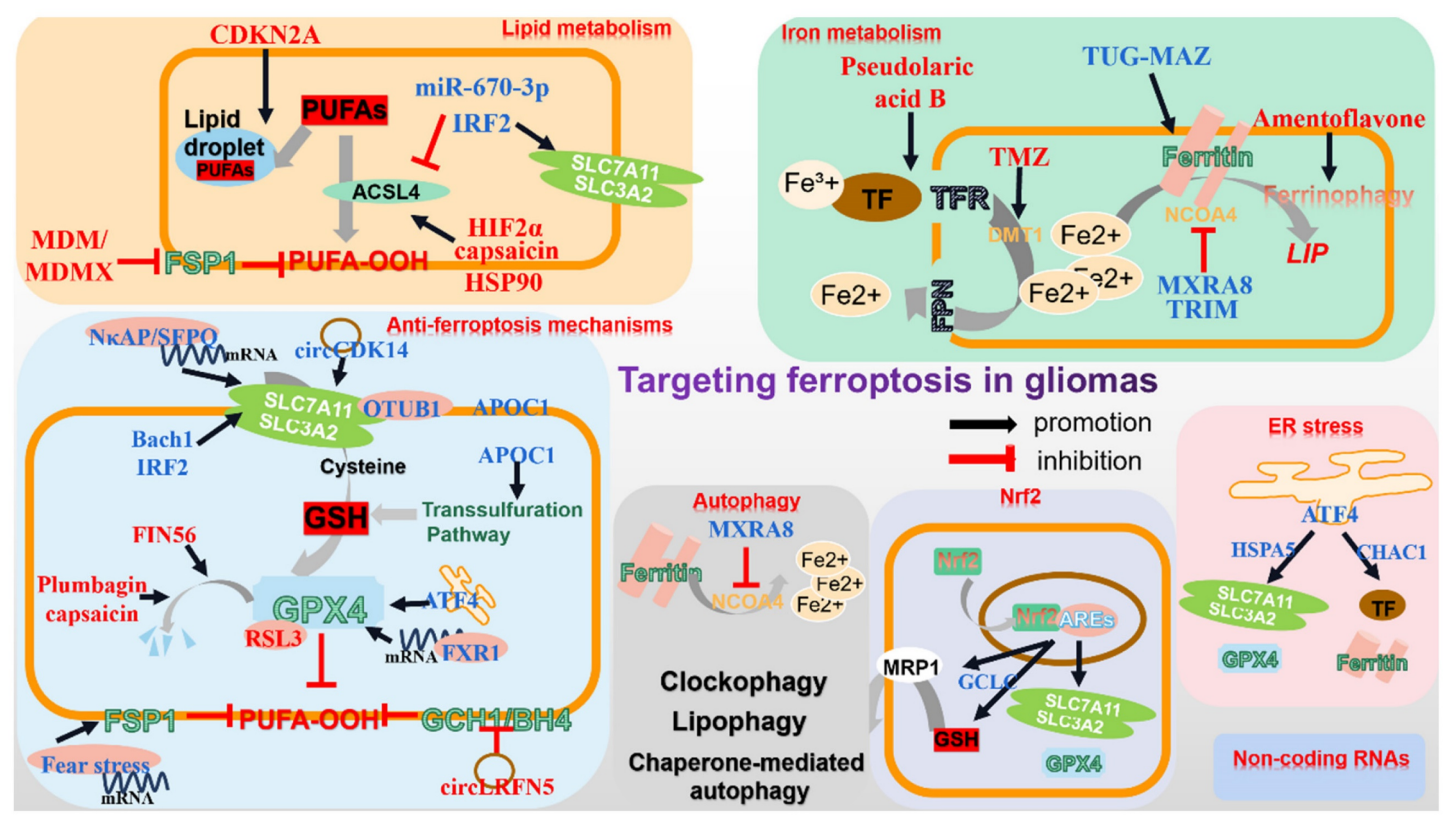
** Targeting different ferroptosis-related pathways in gliomas.** The current research focuses on regulating various ferroptosis-related molecules to induce glioma cell death. In terms of iron and lipid metabolism, elevated intracellular iron content and overwhelming membrane PUFAs enhance ferroptosis susceptibility. Through regulating the mRNA of ferroptosis-related molecules, non-coding RNAs significantly impact glioma progression. Additionally, autophagy, particularly ferritinophagy markedly facilitates ferroptosis. Moreover, ER stress, Nrf2 and classic tumor inhibitor p53 appear to be involved in ferroptosis. All above represent the primary regulators, on which most administration target for ferroptosis ignition and glioma inhibition.

Chaperone-mediated autophagy selectively degrades GPX4 with the assistance of specific molecular chaperones. Erastin can elevate the level of HSP90, a molecular chaperone, which partially accentuates ferroptosis by amplifying GPX4 autophagic degeneration^169^. Beyond that, p62-mediated clockophagy and RAS-related in Brain 7A (RAB7A)-mediated lipophagy are both involved in ferroptosis^170^, ^171^. Despite these, it is puzzling that Mariachiara Buccarelli and colleagues figured out that impairing autophagy with quinacrine augmented GSCs' susceptibility to TMZ by stimulating ferroptosis^172^. Moreover, a lower autophagy level is positively corelated with longer OS time for patients^172^. Overall, autophagy is broadly implicated in ferroptosis and the interaction between them still remains controversial (Fig. 2).

### 3.7 Temozolomide resistance and ferroptosis

Temozolomide represents a first-line drug for glioma treatment by inducing DNA methylation and subsequent replication blockage, but its effectiveness is greatly hampered due to chemotherapy resistance. Along with O6-methylguanine-DNA methyltransferase-mediated DNA repair and abnormal apoptotic signaling, ferroptosis is also identified tightly associated with temozolomide tolerance. On the one hand, TMZ causes glioma cell death partially by facilitating DMT1-related ferroptosis^104^. Furthermore, autophagy inhibition combined with temozolomide generates intensive ferroptosis^172^. On the other hand, ferroptosis has a significant impact on TMZ sensitivity. In details, factors enhancing ferroptosis generally render glioma cells more sensitize to TMZ, whereas compounds and molecular suppressing ferroptosis tend to contribute to TMZ resistance^111^, ^140^. Accordingly, the combination of temozolomide and ferroptosis inducers exhibits a better inhibitory effect on gliomas^120^. The dopamine D2 receptor (DRD2) induced by TMZ is inversely correlated with temozolomide sensitivity in GBM. DRD2 antagonist haloperidol amplifies TMZ-induced ER stress by activating the cAMP/PKA pathway, thereby reinforcing ferritinophagy-mediated ferroptosis and leading to an enhanced antitumor effect^163^. Counterintuitively, some features that limit oxidative stress make temozolomide-tolerant cells susceptible to ferroptosis, which warrants further exploration^149^. Overall, triggering ferroptosis notably heightens the efficacy of temozolomide.

### 3.8 Glioma immune microenvironment and ferroptosis

The interplay between ferroptosis and immune responses remains complicated and vague in gliomas. Generally, immune cells exert antitumor effects partially by igniting ferroptosis in gliomas. For instance, activated CD^8+^ T cells modulate the INFR-STAT1-SLC7A11 axis to trigger tumor cell ferroptosis by secreting IFNγ. In addition, TGFβ released by macrophages mediates the disruption of xCT-GSH-GPX4 axis via SMAD protein, thus triggering lipid peroxidation-mediated tumor cell death^173^, ^174^. The nano-platform containing siPD-L1 markedly eliminates PD-L1 and increases the proportion of effector T cells with tumor killing efficacy. Moreover, the mediators released by ferroptotic cells promote DC cells maturation and subsequent stimulation of the effector T cells^174^. Furthermore, the administration of S-biAb/dEGCG@NPs not only strengthens IFNγ-mediated tumorcidal efficacy by targeting B7-H3 which prevents T cell activation, but also significantly downregulates GPX4, intensely boosting the capacity of immune checkpoint blockage therapy^175^. The advances in bio-nanotechnology shed new light on restraining glioma progression by manipulating tumor ferroptosis and immune responses.

However, only 8% of glioma patients respond effectively to immunotherapy, despite its promising results in many other tumors, which is predominantly attributed to the unique tumor immunosuppressive microenvironment in gliomas. Unconventionally, ferroptosis appears to contribute to immunotherapy ineffectiveness by reestablishing the tumor microenvironment. A recently conducted large cohort study has substantiated that ferroptosis is the most dominant form of PCD in gliomas and its prevalence increases with the grade of gliomas. Large amounts of DAMPs, including cytokines and inflammatory modulators released from glioma cells that undergo ferroptosis, evokes an intense dysfunctional immune response and recruits numerous immune cells^176^. Ferroptosis markedly enhances the abundance of tumor-associated macrophages (TAMs), the most plentiful immune cells in the glioma microenvironment. Mechanistically, high ferritin light chain (FTL) in TAMs promotes M2 polarization through facilitating ferroptosis by inhibiting the expression of iPLA2β, which expedites tumor progression through inducing an immunosuppressive microenvironment, promoting tolerance to anti-PD-L1 therapy, as well as facilitating glioma angiogenesis^177^. Similarly, the immunogenicity generated by ferroptotic tumor cells promotes DC cells maturation while restricting their function. Beyond that, elevated ferroptosis greatly diminishes the oncolytic ability of effector T cells. Other cells, such as cancer-associated fibroblasts, may also play a role in orchestrating glioma ferroptosis^142^. Besides, the Treg cells responsible for immunosuppression are more resistant to ferroptotic stress and partly contribute to tumor progression^178^. Most importantly, ferroptosis appears to be responsible for the ineffectiveness of immune checkpoint blockade treatment in gliomas. It has been indicated that depletion of ferroptosis immensely and synergistically improves the antitumor efficacy of PD-1/PD-L1 blockage by revitalizing the immune microenvironment^176^. Lastly, neutrophils recruited by ferroptotic injury amplify tumor necrosis by triggering ferroptosis in gliomas, which clinically predicts a worse prognosis^17^.

**Figure 3:**
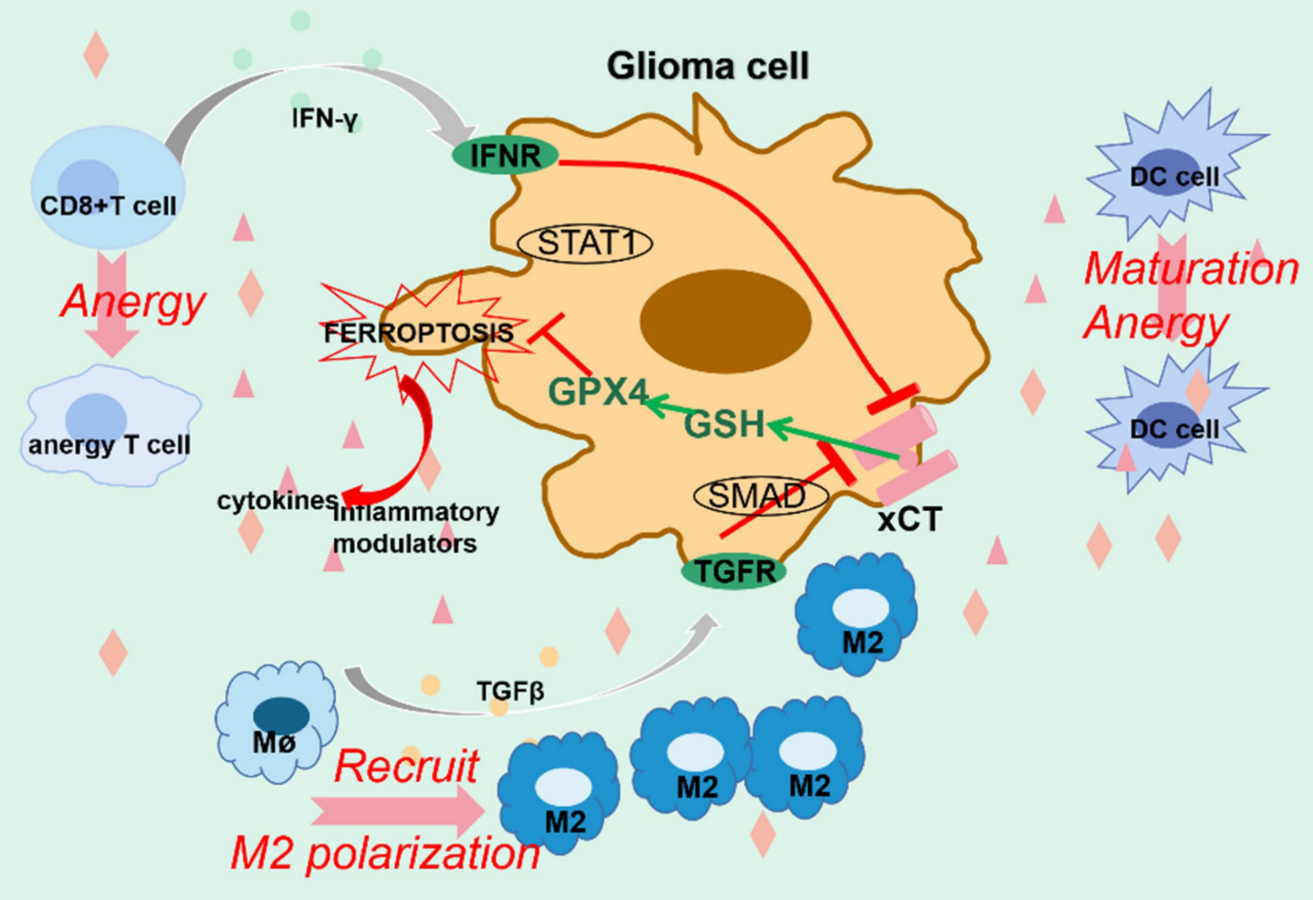
**Ferroptosis and glioma immune microenvironment.** The interplay between ferroptosis and glioma immune microenvironment remains intricate and obscure. For one thing, immune cells partially function to restrain glioma by evoking ferroptotic cell death. For another thing, however, ferroptotic glioma cells release abundant modulators to reestablish the microenvironment, which causes anergy in immune cells and worsens their ability to combat tumor, contributing to tumor progression. Furthermore, enhanced ferroptosis may account for the ineffectiveness of PD-1/PD-L1 blockage in treating gliomas.

In brief, although tumor cell ferroptosis effectively hinders the progression of gliomas, in vivo ferroptosis seems to contrarily promote the advancement of malignant phenotypes by modifying the tumor immune microenvironment. We herein propose reasonable hypotheses potentially counting for the contradictory phenomena. Firstly, mild/moderate ferroptotic damage in the early stage of gliomas may force surviving tumor cells to develop resistance to immune attack. Secondly, plentiful mediators derived from glioma cells undergoing ferroptosis can reestablish the tumor microenvironment, making it inhospitable to tumor-killing cells. Lastly, the immunogenicity generated by ferroptosis cells recruits a large number of immune cells and alters their phenotypes, thus immensely intensifying immunosuppression and dampening the effectiveness of immunotherapy. Still, the challenge lies in optimizing the therapeutic benefits of ferroptosis by addressing a balance between triggering tumor cell death and overcoming the immunosuppressive phenotype in the meanwhile (Fig. 3).

### 3.9 Nano-delivery systems and ferroptosis

Many compounds capable of igniting ferroptosis confront with the obstacles including short circulatory half-life, low BBB penetration and low intratumoral permeability, resulting in poor clinical effect^179^. Advances in nanotechnology have brought solutions to these problems with distinct advantages. The minimal size combined with molecular modifications such as integrins greatly improves the BBB permeability and the efficiency of intratumoral penetration. The iron oxide nanoparticles loaded with paclitaxel (IONP@PTX) drastically retards GBM progression by enhancing autophagy-dependent ferroptosis, where defects of paclitaxel including poor solubility in water and low bioavailability have been greatly overcome^167^. In addition, nano-delivery systems can deliver compounds targeting diverse ferroptosis defenses simultaneously, thus achieving synergistic effect. An engineered exosome-conjugated magnetic nanoparticles possessing the capacity of causing synchronous disintegration of ferroptosis defense axis has been generated. Combining brequinar, a DHODH inhibitor, si-GPX4 and Fe_3_O_4_, the platform results in violent ferroptosis in GBM and yields a markable therapeutic effect. Notably, the platform can be enriched in the brain under local magnetic localization and cross the BBB and selectively target GBM by recognizing the LRP-1 receptor via the assistance of angiopep-2-modified engineered exosomes^113^. Moreover, combining with other antitumor therapies such as sonodynamic therapy is achievable via nano-platforms. The PIOC@CM NPs encapsulating both Fe_3_O_4_ and Ce6 selectively accumulate in the tumor and increase intracellular ROS while decreasing GSH and GPX4 under ultrasonic excitation, ultimately leading to ferroptotic death^180^.

Surprisingly, biomimetic nanomaterials are capable of hindering tumor progression through modulating immune microenvironment. For instance, the delivery system Fe_3_O_4_-siPD-L1@M_-BV2_ significantly boosts the immune response against GBM. For one thing, Fe_3_O_4_ triggers intense ferroptosis in tumor cells. For another thing, the siPD-L1 greatly eliminates PD-L1 and increases the proportion of effector T cells with tumor killing efficacy. Moreover, the mediators released by ferroptotic cells promote DC cells maturation and the effector T cells may eradicate tumor cells by inducing tumor ferroptosis^174^.

In conclusion, strategies based on nanomaterials and ferroptosis have provided new insights into the management of gliomas. Nonetheless, further research is necessary to determine how nanotechnology can be utilized to develop personalized treatment strategies for glioma patients, ultimately achieving precision medicine.

## 4. Future perspectives

Indeed, splendid strides have been achieved in the field of glioma treatment by manipulating ferroptosis of tumor cells to disrupt their progression^181^. However, regarding the clinical application of ferroptosis based strategies at the aim of improving prognosis for glioma patients, several issues are to be addressed. Firstly, multiple ferroptosis defending signals in tumor cells have reciprocal compensatory effects, weakening the anti-cancer effect of a single intervention^79^. Secondly, the interaction between tumor cells and non-tumor cells in the microenvironment is crucial for glioma, and determining the role of prominent non-tumor cells in ferroptosis resistance of glioma cells may provide new therapeutic ideas^182^. In addition, the inhibiting effect of conventional drugs (including synthetic drugs and naturally extracted drugs) on glioma ferroptosis is severely limited by the BBB. Furthermore, immunotherapy is a promising treatment approach in the field of gliomas. However, the relationship between ferroptosis and tumor immune responses is intricate and inexplicit^183^. Briefly, numerous mediators from ferroptotic tumor cells promote the maturation of antigen-presenting cells and the proliferation of effector T cells. On the other hand, these immune cells have been elucidated to exhibit anergy, losing their tumor-killing ability. Furthermore, the modified tumor microenvironment promotes macrophages towards M2 polarization, which in turn contributes to gliomas progression^177^.

In the future, from our standpoint, the clinical application of ferroptosis based strategies are to focus on following aspects. First of all, the establishment of a correlation between cerebrospinal fluid ferroptosis markers and glioma patient outcomes presents an appealing prognostic approach. And the advancement of in vivo imaging technology may enable the direct observation of the regulatory effect of ferroptosis in brain^184^. Additionally, the pursuit of targeting ferroptosis in both glioma and major non-tumor cells within the microenvironment may potentially lead to enhanced therapeutic outcomes. Moreover, considering the varying tolerance level to ferroptosis among different immune cells^178^, it holds great promise to improve the impact of immunotherapy by inducing specific degree of ferroptosis. Furthermore, the nanoplatform-mediated glioma ferroptosis is expected to become a prominent focus in future clinical application due to its significant advantages, including enhanced payload capacity, precise targeting capabilities, and efficient BBB penetration.

## 5. Conclusion

Over the past decades, ferroptosis has been demonstrated morbidly implicated in the progression of gliomas. Furthermore, the unique intrinsic properties make glioma ferroptosis distinct from that in other tumors. Firstly, ferroptosis has been identified as the most enriched PCD in gliomas, suggesting that intervening in ferroptosis in gliomas may produce more magnificent anti-cancer effects than in other tumors^176^. The brain, being the organ with the highest lipid content, particularly rich in PUFAs, exhibits a significant susceptibility to the Fenton reaction^185^. Moreover, gliomas exhibit a high dependency on glutamine uptake, whose catabolite glutamate subsequently reduces their vulnerability to ferroptosis by exchanging with extracellular cystine^186^. Nonetheless, mounting studies have indicated that although ferroptosis can induce pronounced glioma impediment, the ferroptotic cells paradoxically create a favorable niche for the survival of residual tumor cells^17^, ^176^. With the landscape of ferroptosis in gliomas being increasingly well-characterized, and various approaches of inducing ferroptosis to suppress tumors cumulatively proven effective^86^, we point it out here various unsolved challenges to be addressed in this field.

Ferroptosis is intricately intertwined with other biological processes, which have been unveiled to counteract the anticancer effect of ferroptosis, forming an intricate signal network^14^, ^187^. For example, while causing glioma ferroptosis, DHA also activates the ER stress, which in turn alleviates the extent of lipid peroxidation^124^. Furthermore, although ferroptosis is an autophagy-dependent PCD, inhibition of autophagy unexpectedly intensifies ferroptosis and raises temozolomide sensitivity^172^. Moreover, the interaction among mitochondrial homeostasis, lysosome and Golgi stress and ferroptosis is also noteworthy^14^.

Ferroptosis is closely associated with temozolomide efficacy. Generally, temozolomide may function partly by inducing ferroptosis in gliomas^104^, whereas ferroptosis in turn boosts temozolomide sensitivity. It is unsatisfactory to merely trigger gliomas ferroptosis. In the future, for better clinical outcomes, exploring the usage of clinically available ferroptosis inducers in combination with temozolomide could be a new promising strategy. Considering the sensitivity of normal brain cells, especially neurons, to ferroptosis, these inducers are expected to possess high BBB penetration, mild to moderate death-inducing effects, and high glioma targeting, which calls for further clinical investigation.

Ferroptosis is excellent in repressing gliomas. Unfortunately, surviving tumor cells may acquire ferroptosis tolerance by developing various mechanisms^176^. Apparently, it is urgent to elucidate the specific mechanisms by which residual tumor cells acquire ferroptosis resistance, which will provide the potential for the eventual elimination of gliomas. Particularly, the occurrence of ferroptosis in other components of the tumor microenvironment, including stromal cells, astrocytes, immune cells, deserves attention, which may open new avenues in glioma therapy^142^. However, there has not been conclusion about which non-tumor cells are most significant for glioma ferroptosis resistance. Moreover, it is not plausible at present to target specific cell populations to induce cell death. In the future, this may be achievable via nano-delivery systems.

The significance of glioma ferroptosis for immunotherapy efficacy deserves special attention. In vitro, ferroptotic cell death significantly inhibited glioma progression. Nonetheless, in vivo, abundant ferroptosis has been unconventionally elucidated to contribute to the frustration of immunotherapy in gliomas^176^. What determines the differences in outcomes of glioma ferroptosis in vivo and in vitro? And more meaningfully, it remains an open question how to promote the effect of emerging immunotherapy, represented by PD-1/PD-L1 blockade, by manipulating ferroptosis, and how to strike a balance between the anti-tumor and pro-tumor effects of ferroptosis.

**Figure 4:**
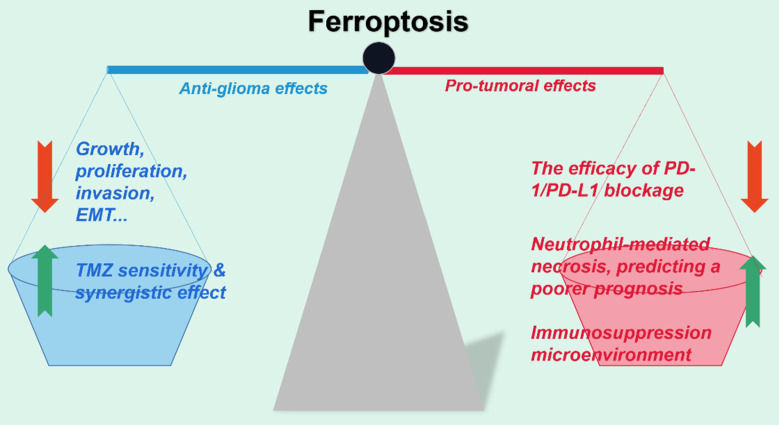
**Optimize the therapeutic efficacy of ferroptosis on gliomas.** Ferroptosis induced by various administrations notably impedes glioma viability, proliferation, invasion and other malignant phenotypes in vitro. However, studies have also regarded ferroptosis as the accomplice of the failure of immunotherapy in gliomas in vivo and the poor prognosis of patients. Therefore, figuring out the optimized strategy by balancing both the anti-tumor and pro-tumor effect of ferroptosis in gliomas calls for further investigation.

In conclusion, ferroptosis has been confirmed to play a significant role in a series of pathological processes of gliomas, including proliferation, invasion, metastasis and drug resistance. The induction of ferroptosis in tumor cells has also been identified an effective tumor suppressor. Nonetheless, further exploration is necessary, considering the underlying details and the obstacles mentioned above. More significantly, given that most of the existing studies have been conducted on cells and animals, the differences between animals and human are to be noted, highlighting the urgent need for more clinical studies for better clinical application. Furthermore, the field of biomedical engineering represented by nanotechnology may provide a promising approach for precision medicine in gliomas^188^ (Fig. 4).

## Abbreviations

PCD: programmed cell death
OS: overall survival
GBM: glioblastoma multiforme
PUFA: polyunsaturated fatty acid
ROS: reactive oxygen species
ACSL4: acyl-CoA synthetase long-chain family member 4
LPCAT3: lysophosphatidylcholine acyltransferase 3
iPLA2β: phospholipase A2 group VI
LOXs: lipoxygenase
PEBP1: phosphatidylethanolamine binding protein 1
NOXs: NADPH oxidase
POR: P450 (cytochrome) oxidoreductase
TF: transferrin
TFR: transferrin receptor
STEAP3: six-transmembrane epithelial antigen of prostate 3
FPN: ferroportin
LIP: labile iron pool
IREB2: Iron responsive element binding protein 2
NCOA4: nuclear receptor coactivator 4
GPX4: glutathione peroxidase 4
SLC7A11: solute carrier family 7 member 11
SLC3A2: regulatory subunit solute carrier family 3 member 2
GSH: glutathione
GCL: glutamate cysteine ligase
GCS: glutamylcysteine synthetase
OTUB1: OTU domain-containing ubiquitin aldehyde-binding proteins otubain 1
TCF4: transcription factor 4
FSP1: ferroptosis suppressor protein 1
RTA: radical-trapping antioxidant
DHODH: dihydroorotate dehydrogenase
GCH1: GTP cyclohydrolase 1
BH4/BH2: tetrahydrobiopterin/dihydrobiopterin
DHFR: dihydrofolate reductase
IL4i1: interleukin-4-induced-1
IDO1: indoleamine2,3-dioxygenase1
IRF2: interferon regulatory factor 2
HSP90: heat-shock protein 90
MDM/MDMX: Murine Double Minute 2/X
CDKN2A: cyclin dependent kinase inhibitor 2A
TAGs: triacylglycerides
DMT1: divalent metal transporter 1
DHA: Dihydroartemisinin
MAZ: Myc-associated zinc finger protein
FTH: heavy ferritin chain
TRIM: Tripartitemotif
MXRA8: matrix remodeling-associated protein 8
ALDH1a3: Aldehyde Dehydrogenase 1 Family Member A3
NκAP: NF-κB activating protein
SFPQ: splicing factor proline and glutamine-rich
Bach1: BTB and CNC homology 1
PDGFRA: platelet derived growth factor receptor alpha
TXNDC12: thioredoxin domain protein 12
FXR1: fragile X mental retardation 1
APOC1: Apolipoprotein C1
PRRX2: paired related homeobox 2
ADAR: adenosine deaminase acting on RNA
FANCD2: fanconi anemia complementation group D2
HSF1: heat shock factor 1
ZFP36: zinc finger protein 36
SRSF1: serine/arginine splicing factor 1
ITGB8: integrin subunit beta 8
GCLC: glutamate-cysteine cigase catalytic subunit
ABCC1: adenosine triphosphate binding cassette subfamily C member 1
CHAC1: ChaC glutathione-specific gamma-glutamylcyclotransferase 1
RAB7A: RAS-related in Brain 7A
DRD2: dopamine D2 receptor
TAMs: tumor-associated macrophages
FTL: high ferritin light chain
